# Quantification of an alpha flux based radiological dose from seasonal exposure to ^222^Rn, ^220^Rn and their different EEC species

**DOI:** 10.1038/s41598-019-38871-6

**Published:** 2019-02-21

**Authors:** Pargin Bangotra, Rohit Mehra, Rajan Jakhu, Pragya Pandit, Mukesh Prasad

**Affiliations:** 1 Radiation Physics Laboratory, Dr. B.R. Ambedkar National Institute of Technology, Punjab, India; 2 Atomic Minerals Directorate For Exploration and Research, New Delhi, India; 3 Department of Physics, H.N.B. Garhwal University, Badshahi Thaul Campus, Tehri Garhwal, India

## Abstract

This study summarizes the seasonal experimental data on the activity concentrations of indoor ^222^Rn (Radon), ^220^Rn (Thoron) and their progeny in Mansa and Muktsar districts of Punjab (India) using LR-115 solid state nuclear track detector based time integrated pin-hole cup dosimeters and deposition based progeny sensors for the assessment of radiological dose. The indoor ^222^Rn concentration was observed higher in the rainy and winter seasons while ^220^Rn concentration was observed higher in the winter season. However, Equilibrium Equivalent Concentrations (EECs) of ^222^Rn and ^220^Rn exhibited distinct seasonal behaviour unlike their parent nuclides. The average equilibrium factors for ^222^Rn (F_Rn_) and ^220^Rn (F_Tn_) were found 0.47 ± 0.1 and 0.05 ± 0.01, respectively. The annual arithmetic means of unattached fractions of ^222^Rn (${f}_{p}^{Rn}$) and ^220^Rn (${f}_{p}^{Tn}$) were found to be 0.09 ± 0.02 and 0.10 ± 0.02, respectively. The attachment rate (*X*_*Rn*_) and attachment rate coefficients (*β*) of ^222^Rn progeny were also calculated to understand the proper behaviour of progeny species in the region. A new alpha flux based technique has been proposed and used for the assessment of absorbed dose rate and annual effective dose rate for radiation protection purpose.

## Introduction

^222^Rn and ^220^Rn are naturally occurring radionuclides formed within the decay series of ^238^U and ^232^Th, respectively. Exhalation and emanation processes are responsible for the migration of ^222^Rn and ^220^Rn from its parent radionuclides (present in soil or building material) to indoor environment and thereby contributing radiological dose to mankind^1–5^. In general, radioactive aerosols can be categorized as radioactive nuclides of cosmogenic origin (^7^Be, ^22^Na and ^32^P), ^222^Rn and ^220^Rn decay products aerosols (^218^Po, ^216^Po, ^214^Po and ^212^Po), aerosols associated with the high-energy accelerators (^24^Na, ^52^Mn and ^7^Be) and Fission product radionuclide aerosols (^89^Sr, ^137^Cs, ^131^I and ^140^Ba). Among numerous sources of manmade and natural background radiation, a major part of the dose comes from α- emissions from decay products of ^222^Rn and ^220^Rn (1.26 mSv out of 2.4 mSv). These radionuclides tend to attach themselves to aerosol particles and then get deposited in the respiratory tract^6–8^. Equilibrium factors of ^222^Rn (F_Rn_) and ^220^Rn (F_Tn_) have a vital role in dose assessment. In umpteen scientific reports and manuscripts, the F_Rn_ and F_Th_ in the indoor environment have often been reported to be 0.4 (for ^222^Rn) and 0.1 for ^220^Rn, however equilibrium factor (*F*) is varies with surrounding conditions^6^. The attached and unattached equilibrium equivalent concentrations (EECs), unattached fraction (*f*_*p*_), attachment rate (*X*), aerosol concentration (Z) and dose conversion factors (DCFs) are other weighty parameters in lung dose assessment. The unattached fraction has higher tendency than attached fraction to absorb faster in the blood^9^. For a significant analysis, it is imperative to take into account all these parameters with appropriate seasonal behaviour of ^222^Rn, ^220^Rn and their progeny for the assessment of annual effective dose. The seasonal variations of these parameters are highly influenced by geology and climate of particular area. In present investigation, an effort is made to give a detailed analysis of geology, seasonal variations and climatic conditions of the studied area.

In Indian scenario, the seasonal variation data of *F*, *f*_*p*_, *X* and EECs of ^222^Rn and ^220^Rn are very scanty and negligible emphasis has been given on these parameters in ^222^Rn/^220^Rn studies. The ^220^Rn measurements were also neglected in the past studies due to the assumption of small contribution of ^220^Rn to effective radiological dose. However, recent studies in some countries have unveiled that this assumption may not be entirely correct and ^220^Rn can still be a hazard since its progeny ^212^Pb with a half - life of 10.6 h can accumulate to significant levels in breathing air^10,11^. In this study, all parameters (*X*, *f*_*p*_, F_Rn_, F_Tn_ and Z) that influence the levels of ^222^Rn and ^220^Rn in an indoor environment have been evaluated. In the present work, an attempt has been made to study the seasonal variations of ^222^Rn, ^220^Rn and their EEC species along with the effects and correlation of divergent parameters in dose assessment.

In former studies, the discrepancy of dose conversion factors (DCFs) between epidemiological and dosimetric approach has not been resolved due to their different origins and sources. The dosimetric approach followed the path of Human Respiratory Tract Model (HRTM) with innumerable parameters as breathing frequency, tidal volume, aerosol parameters, unattached fraction and clearance rate. However, epidemiological approach used the data of atomic bomb survival, nuclear accident survival and uranium mine studies. The ICRP initiated with the dosimetric approach and considered the epithelium of the lung as a critical tissue for ^222^Rn exposure^12^. In ICRP publication 26, committee distributed the tissue weighting factors for different tissues or organ in order to estimate the overall risk for a whole body irradiation^13^. A tissue weighting factor of 0.12 has been reserved for lungs. Instead of considering the tissue weighting factor, ICRP changed the radiation weighting factor of an alpha particle from 10 to 20^13,14^. The UNSCEAR committee has also adopted the path of dosimetric approach (based on radiation weighting factor and tissue weighting factor for alpha exposure) with different indoor and outdoor occupancy factors^15^. In 1993, ICRP switched to the epidemiology approach of ^222^Rn exposure in mines (due to less uncertainty) and later biokinetic model (HRTM) for ^222^Rn gas has been developed so that effective doses arising from the inhalation of ^222^Rn gas can be calculated^16–18^. In ICRP publication 115, the commission has concluded that ^222^Rn and its progeny should be treated in the same way as any other radionuclide within the system of protection and proposed that doses from ^222^Rn and its progeny should be calculated using ICRP biokinetic and HRTM (in order to calculate the doses to either organs or lungs)^19^. Further, ICRP revised the HRTM with major changes made to relate the clearance of deposited material by both particle transport and absorption into blood and provided new recommendation of data on systemic biokinetics, inhalation and ingestion for most of the elements^20,21^. In the present manuscript, a different and direct alpha flux-based approach has been used to estimate the radiological dose from ^222^Rn exposure.

## Geology, Climate and Seasons of Studied Area

**Figure 1:**
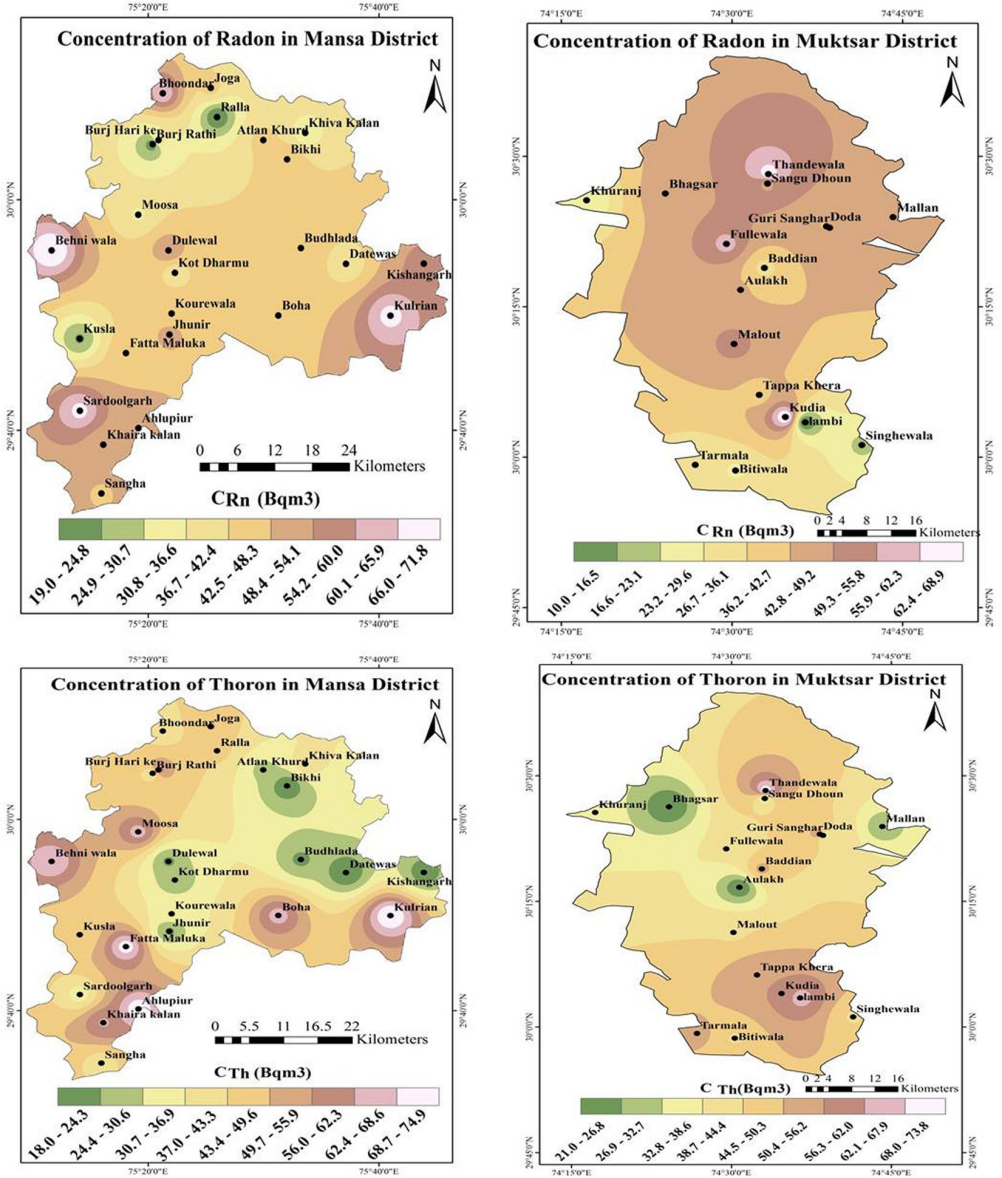
Map of the investigated area of Punjab (India).

Punjab is in north western India and has an area of 50, 362 square kilometers. Geologically, Punjab is formed by alluvial deposits of various rivers flowing in the region. The rocks of Aravalli – Delhi subgroup and the Malani igneous suite comprising greywacke, Ortho – quartzite carbonate sediments, calcareous shales and slates, high heat producing granites and felsites form the basement in the region^22–25^. The scattered outcrops of the Aravali- Delhi Subgroup occur at Tosham (Haryana) just south of the study area i.e. Mansa and Muktsar Districts of Punjab (India) as shown in Fig. 1. The other geological parameters, area and population are given in Table 1 ^26,27^.

**Table 1:** Area, Population and average rainfall in Mansa and Muktsar districts.

District	Area	Population	Average Rainfall (mm)		
			March–June	July–October	November–Feburary
Mansa	2171	408732	86.7	143.1	24.5
Muktsar	2615	475622	154	414.8	15.5

The climate of Punjab is determined by the extreme hot and cold conditions. The Himalayas in North, Deserts of Rajasthan in south, three rivers and famous Indian monsoon influencing the climate and environment of Punjab. The seasons of Punjab can be categorized into summer, rainy, winter, pre -summer and post monsoon seasons. In Punjab, the summer season commences at the start of March. Punjab’s rainy season begins in last week of June monsoon. Three quarters of the total rainfall is concentrated during the three months of southwest monsoon winds and the rest comes during the winter months. There is a wide difference in the amount of rainfall experienced in east and west Punjab. The monthly average rainfall in the studied area has been shown in Table 1 and it has been seen that gap between October and November is a transitional period between monsoon and winter^26,27^. The minimum temperature in studied area has been recorded in months of January – February and maximum in the month of June. The maximum temperature usually occurs in the months of May - June and during this period temperature remains greater than the 40–45 °C in studied area.

## Materials and Methodology

The seasonal behaviour of ^222^Rn, ^220^Rn, unattached fraction and equilibrium factors of ^222^Rn and ^220^Rn have been studied in 42 villages of Mansa and Muktsar districts of Punjab (India). The acquired data points were processed on computer using IDW (Inverse Distance Weighted) algorithm on Arc map GIS (Geographical Information System) 10.3 software^28^. The months of March to mid-June as summer, Second half June to mid-October as rainy and Second half October to February as winter were taken for the seasonal estimation of ^222^Rn, ^220^Rn and their daughter products^27^. The ^222^Rn/^220^Rn based dosimeters and DRPS/DTPS were suspended in the way to 20 cm away from the adjacent walls.

### Estimation of concentration of ^222^Rn and ^220^Rn

**Figure 2:**
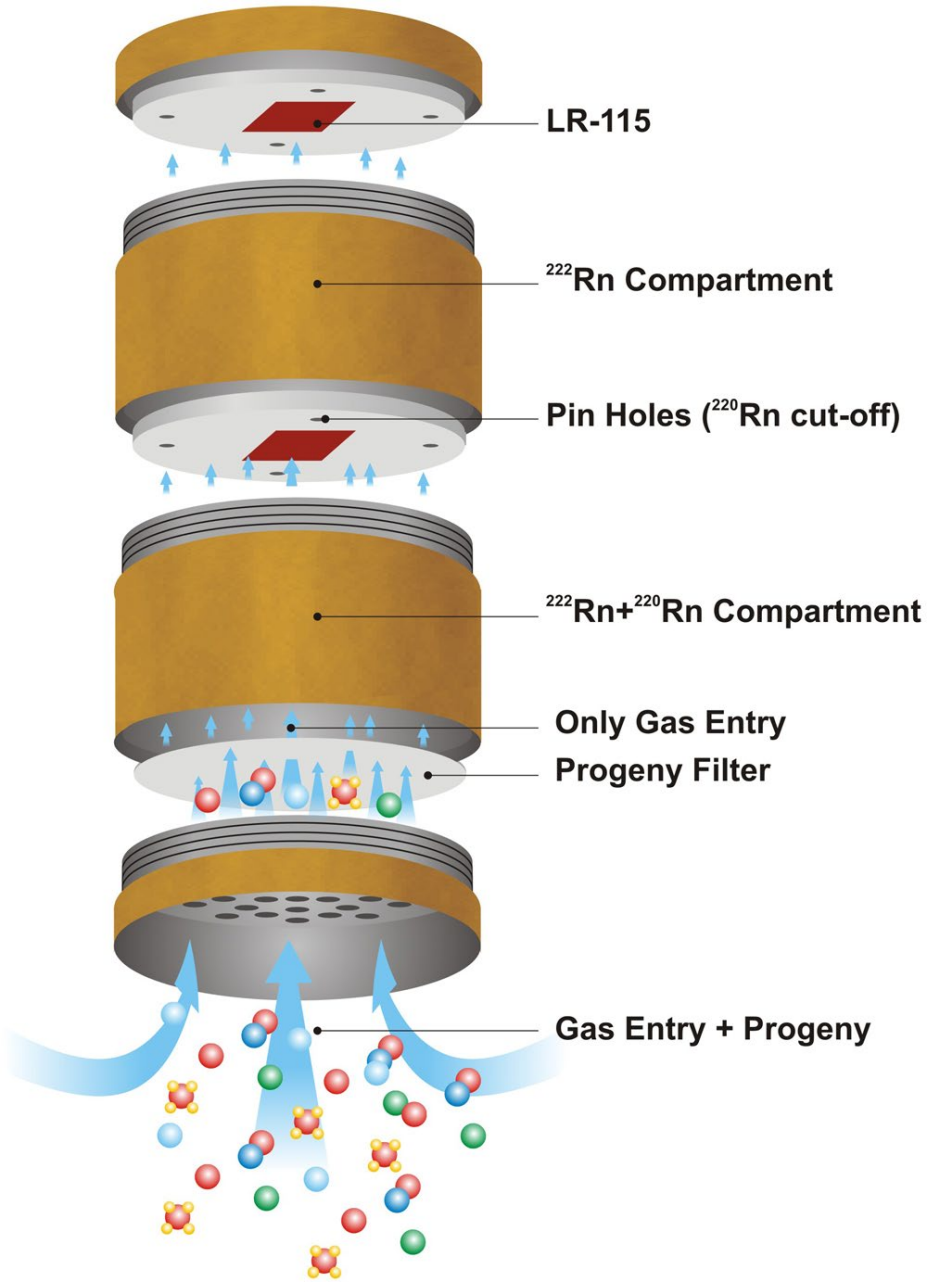
Single entry Pin Hole Cup Dosimeter.

The ^222^Rn and ^220^Rn concentrations in the air were estimated by using a pin hole-cup dosimeter (Fig. 2). The gas enters through the bottom of the dosimeters in the lower chamber (^222^Rn + ^220^Rn chamber) and diffuses to upper chamber (^222^Rn chamber) through four pin holes (2 mm length and 1 mm diameter). The glass fiber filter (0.60 µm) paper has been used to stop the entry of progeny nuclides into the chamber as shown in Fig. 2. LR- 115 detector films (3 × 3 cm^2^) have been installed in both chambers of the dosimeter. The chambers are of cylindrical shape having a length of 4.1 cm and radius 3.1 cm. The dosimeters were deployed in the indoor environment for different seasons of a year. After stipulated time of exposure, LR-115 films were retrieved, chemically etched (2.5 N NaOH solution at 60 °C for 90 minutes) and track densities on LR-115 detectors were counted using the spark counter. The concentrations of ^222^Rn $\{{{\rm{C}}}_{{222}_{{\rm{Rn}}}}({\rm{Bq}}\,{{\rm{m}}}^{-3})\}$ and ^220^Rn ${\{C}_{{220}_{{\rm{Rn}}}}({\rm{Bq}}\,{{\rm{m}}}^{-3})\}$ gases were estimated by using Eqs (1) and (2) ^29^.

1a $${C}_{{222}_{Rn}}(Bq\,{m}^{-3})=\frac{{T}_{R,1}-B}{\,t\,\times \,{k}_{R,1}}$$

1b $${C}_{{222}_{Rn}}(Bq\,{m}^{-3})=\frac{{T}_{R,2}-B}{\,t\,\times \,{k}_{R,2}}$$

2a $${C}_{{220}_{Rn}}(Bq\,{m}^{-3})=\frac{{T}_{T,1}-B}{\,t\,\times \,{k}_{T,1}}$$

2b $${C}_{{220}_{Rn}}(Bq\,{m}^{-3})=\frac{{T}_{T,2}-B}{\,t\,\times \,{k}_{T,2}}$$

where, k_R,1_ {0.0172 Tracks cm^−2^ (Bq m^−3^ d)^−1^}, k_R,2_ {0.0170 Tracks cm^−2^ (Bq m^−3^ d)^−1^}, k_T,1_ {0.010 Tracks cm^−2^ (Bq m^−3^ d)^−1^} and k_T,2_ {0.00052 Tracks cm^−2^ (Bq m^−3^ d)^−1^} are the calibration factors of ^222^Rn and ^220^Rn in the ‘^222^Rn + ^220^Rn’ and ‘^222^Rn’ chamber, respectively. T_R,1_, T_R,2_, T_T,1_ and T_T,2_ are the track densities (Tracks cm^−2^) in LR-115 detectors for the ‘^222^Rn + ^220^Rn’ and ‘^222^Rn’ chamber for ^222^Rn and ^220^Rn exposure respectively. B is the background track density in unexposed LR-115 detector.

### Estimation of Equilibrium Equivalent Concentrations of ^222^Rn and ^220^Rn (EERC/EETC)

**Figure 3:**
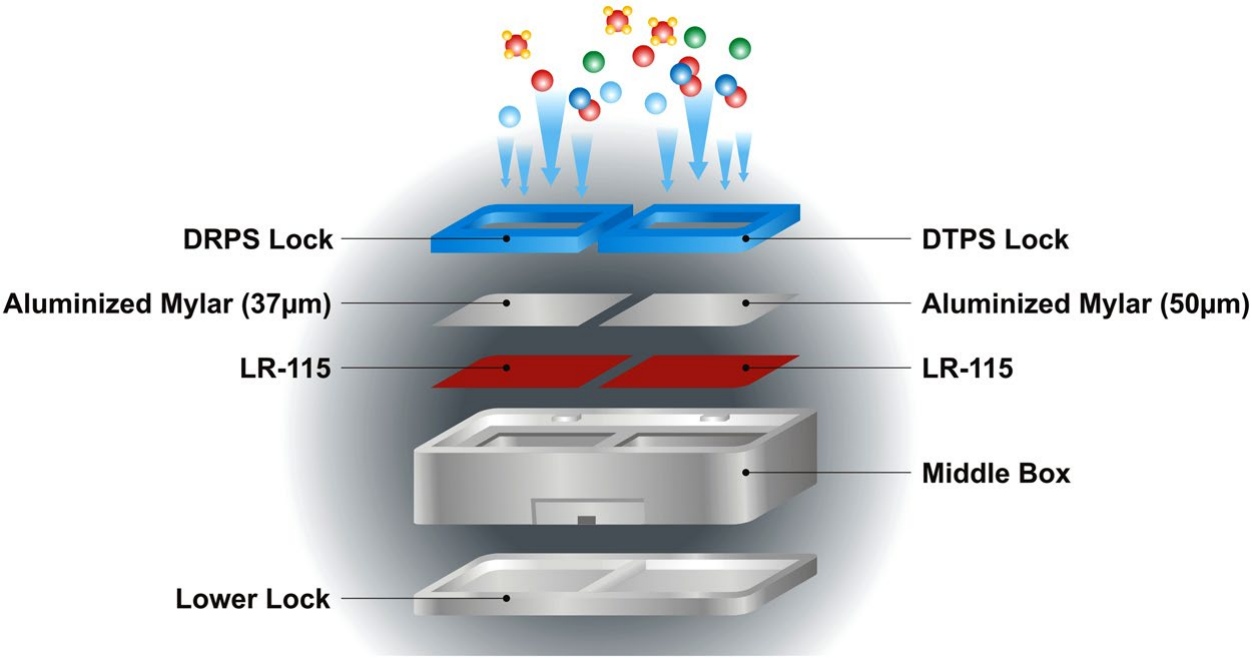
DRPS/ DTPS Sensor.

**Figure 4:**
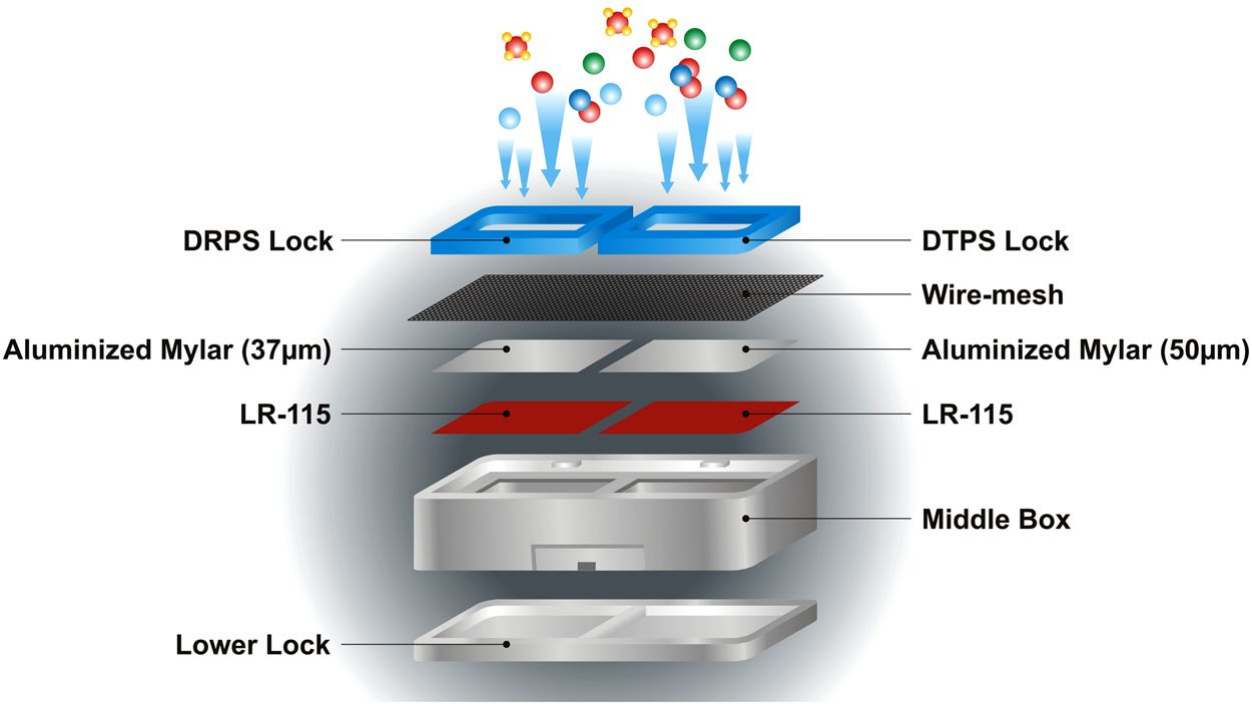
Wire mesh Capped DRPS/DTPS Sensor.

The total unattached and attached EECs of ^222^Rn and ^220^Rn have been measured by DRPS and DTPS progeny sensors as shown in Fig. 3. Both DRPS and DTPS element was made up of LR- 115 (2.5 × 2.5 cm^2^) mounted with absorbers of appropriate thickness as shown in Fig. 3. The EEC has been estimated from the tracks registered on LR-115 films using sensitivity factors used in Eqs (3) and (4) ^30–32^. The coarse (attached) fraction of progeny concentration was measured by the wire-mesh capped DTPS/DRPS (mounted with 200 mesh type wire screens) as shown in Fig. 4.

3 $$EETC(Bq\,{m}^{-3})=\frac{{T}_{T}-\,B}{t\times {S}_{T}}$$

4 $$EERC(Bq{m}^{-3})=\frac{{T}_{R}-\,B-({S}_{{T}^{\text{'}}}\times EETC(Bq{m}^{-3}))}{t\times {S}_{R}}$$

S_T’,_ S_T_, and S_R_ are sensitivity factors of ^220^Rn progeny [0.09 Tracks cm^−2^ d^−1^{EERC (Bq m^−3^)}^−1^] in DRPS, ^220^Rn progeny [0.94 Tracks cm^−2^ d^−1^{EERC (Bq m^−3^)}^−1^] in DTPS and ^222^Rn progeny [0.09 Tracks cm^−2^ d^−1^{EERC (Bq m^−3^)}^−1^] in DRPS, respectively. t is the exposure time period. B, T_T_ and T_R_ are the track densities estimated in unexposed background, DTPS and DRPS LR-115 films respectively. In wire-mesh capped progeny sensors, the sensitivity factors of ^220^Rn and ^222^Rn progeny are replaced by 0.33 Tracks cm^−2^ d^−1^{(EETC (Bq m^−3^)}^−1^ and 0.04 Tracks cm^−2^ d^−1^ {EERC (Bq m^−3^)}^−1^ ^32^. Deposition based ^222^Rn progeny sensor and ^220^Rn progeny sensor have minimum detection limit of 1.0 Bq m^−3^ and 0.1 Bq m^−3^ respectively^33^.

## Theoretical Formalism

### Attachment Rate and attachment coefficient

The activity size distribution *f*_*a*_(*d*) of the radionuclides and the number size distribution *Z*(*d*) strongly depend on attachment process and a function of particle size. The following expression can be used to evaluate *f*_*a*_(*d*)^34^.

5 $${f}_{a}(d)=\beta (d)\frac{Z(d)}{{X}_{Rn}}$$

The function *β*(*d*) is attachment coefficient given by

6 $$\beta (d)=\frac{2\pi {D}_{0}d}{\frac{8{D}_{0}}{d{v}_{0}}+\,\frac{d}{2\delta }}$$

where, *D*_0_ = 6.8 × 10^−2^ *cm*^2^*s*^−1^ is diffusion coefficient, *v*_0_ = 1.72 × 10^4^ *cm* *s*^−1^ is mean thermal velocity and *δ* = *d*/2 + *l*_0_ with *l*_0_ = 4.9 × 10^−6^ *cm* is mean free path of the unattached decay product cluster^34^. Function *Z*(*d*) was normalized to unit aerosol concentration (*N*_0_) as:

7 $$\int Z({d}_{{\rm{p}}}){\rm{d}}{d}_{{\rm{p}}}=1$$

Here, *β*(*d*) is the attachment coefficient of attached ^222^Rn progeny to aerosol and *Z*(*d*) represents aerosol concentration (*cm*^−3^). In the present investigation, the attachment rate of ^222^Rn (*X*_*Rn*_) has been calculated from the measurement of unattached and attached activity concentrations of ^214^Po as follows:

8 $${X}_{Rn}=\frac{EER{C}_{A}}{EER{C}_{U}}\times {\lambda }_{Rn}$$

The average attachment coefficient is defined as to be ratio of the attachment rate and aerosol number concentration given as

9 $$\beta =\frac{{X}_{Rn}}{{N}_{0}}=\frac{{\int }^{}\beta ({d}_{{\rm{p}}})\,Z({d}_{{\rm{p}}}){\rm{d}}{d}_{{\rm{p}}}}{{\int }^{}Z({d}_{{\rm{p}}}){\rm{d}}{d}_{{\rm{p}}}}$$

10 $${X}_{Rn}={\int }_{0}^{\infty }\,\beta (d)Z(d){\rm{d}}d$$

where, EERC_A_, EERC_U_ and λ_Rn_ are attached activity concentration, unattached activity concentration and decay constant of ^214^Po, respectively.

### Equilibrium Factor (*F*_*Rn*_, *F*_*Tn*_)

Equilibrium factor for both ^222^Rn and ^220^Rn has been calculated using following equations:

11 $${F}_{Rn}=\frac{EER{C}_{A+U}}{{C}_{Rn}}$$

11a $${F}_{Tn}=\,\frac{EET{C}_{A+U}}{{C}_{Tn}}$$

*F*_*Rn*_ and *F*_*Tn*_ are equilibrium factor for ^222^Rn and ^220^Rn, respectively. The EERC_A+U_ and EETC_A+U_ are total (attached + unattached) equilibrium equivalent concentrations of ^222^Rn and ^220^Rn progeny, respectively.

## Results and Discussion

### Seasonal variation of ^222^Rn and ^220^Rn

**Figure 5:**
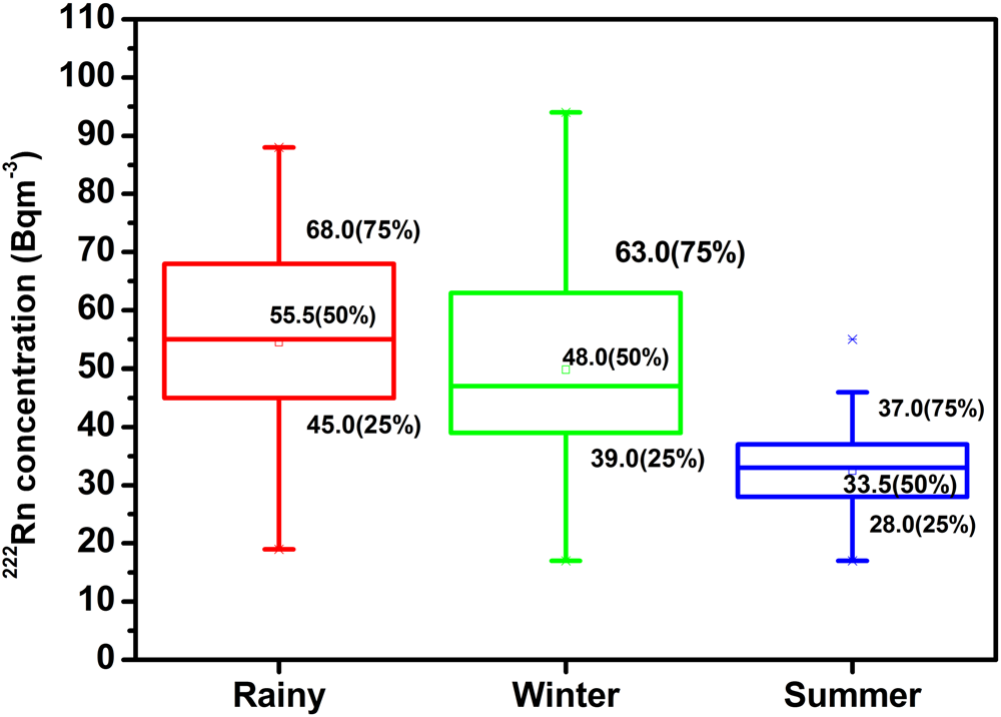
Seasonal Variation of ^222^Rn.

**Figure 6:**
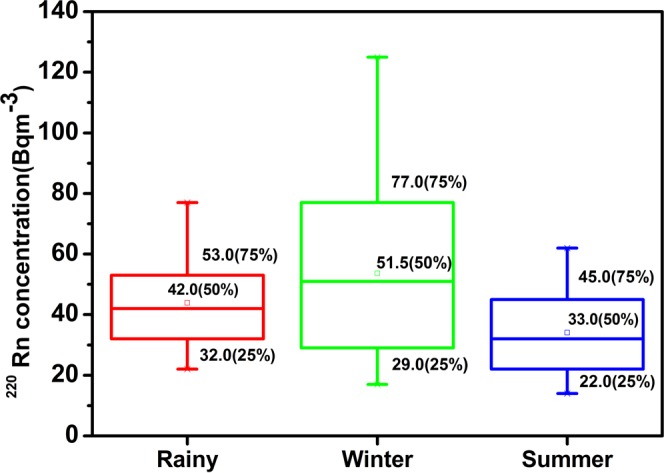
Seasonal Variation of ^220^Rn.

Prior to statistical analysis, the reliability of the data has been tested. The observed value of Kronbach alpha (=0.816 > 0.7) revealed that the data was statistically significant. Few outliers (cases with standardized residual greater than ±3 standard deviations) are found (Table 2) and further data have been tested for normality (Kolmogorov-Smirnov). A robust statistics has been used to study the descriptive analysis of the different radiological parameters and recorded the lower quartile (25%), median quartile (50%) and upper quartile (75%) using tukey’s Hinges and visualized by box - whisker plots as depicted in Figs 5 and 6. The mean, trimmed mean (5%), Inter-quartile range (IQR) corresponding to the dispersion, standard deviation (S.D.) and variance are also reported in Table 2. The asymmetry and tailness of the distribution is indicated by the skewness (S_k_) and kurtosis (K) of the data. Relative variability has been analyzed by the absolute median deviation (AMD). All the above tests have been performed at 95% confidence interval and no missing values have been observed.

**Table 2:** Different statistical parameters of indoor ^222^Rn and ^220^Rn in Mansa and Muktsar districts (42 locations).

Parameter	Season	Mean	Median	GM	Variance	S.D	Min	Max	Range	IQR	S_k_	K	AMD
*C*_*Rn*_ (Bq m^−3^)	Winter	50	48	46	380.6	19.5	17	94	77	24.75	0.419	−0.291	11
	Summer	32	34	31	71.4	8.5	17	55	38	10	−0.008	0.087	5
	Rainy	54	56	51	299.4	17.3	19	88	69	23.75	−0.206	−0.424	11.5
*C*_*Tn*_ (Bq m^−3^)	Winter	54	52	46	797.6	28.2	17	125	108	49	0.615	−0.515	10.5
	Summer	34	33	32	167.6	13.0	14	62	48	23.25	0.214	−0.924	12
	Rainy	44	42	41	221.6	14.9	22	77	55	22.25	0.494	−0.525	10.5
EERC_(A+U)_ (Bq m^−3^)	Winter	24.1	21.5	22.3	107.1	10.4	9	57	48	12	1.48	2.48	5.5
	Summer	15.1	14.0	14.4	23.6	4.9	8	26	18	7.25	0.523	−0.776	3.5
	Rainy	21.9	19.0	20.3	84.6	9.2	11	50	39	10.5	1.32	1.47	5
EERC_A_ (Bq m^−3^)	Winter	22.4	20.0	20.6	103.3	10.2	8	55	47	12	1.52	2.6	5.5
	Summer	13.5	12.5	12.8	21.1	4.6	7	24	17	7.5	0.574	−0.692	3.5
	Rainy	20.0	20.0	18.4	77.4	8.8	10	47	37	10.5	1.32	1.49	4.5
EERC_U_ (Bq m^−3^)	Winter	1.7	2.0	1.5	0.62	0.79	1	4	3	1	1.31	1.93	1
	Summer	1.6	2.0	1.5	0.30	0.54	1	3	2	1	0.08	−1.04	0
	Rainy	1.9	2.0	1.8	0.51	0.71	1	4	3	1	0.53	0.557	0
EETC _(A+U)_ (Bq m^−3^)	Winter	1.8	1.7	1.6	0.482	0.69	0.5	3.5	3	0.95	0.596	−0.069	0.5
	Summer	1.5	1.4	1.4	0.269	0.52	0.7	3	2.3	0.7	0.904	0.802	0.35
	Rainy	1.7	1.6	1.6	0.181	0.43	1.1	2.8	1.7	0.6	0.741	0.308	0.3
EETC_A_ (Bq m^−3^)	Winter	1.6	1.6	1.5	0.414	0.64	0.5	3.2	2.7	0.93	0.64	−0.11	0.45
	Summer	1.3	1.2	1.2	0.225	0.47	0.6	2.7	2.1	0.63	1.02	1.2	0.3
	Rainy	1.5	1.5	1.4	0.168	0.41	0.9	2.6	1.7	0.6	0.677	0.204	0.25
EETC_U_ (Bq m^−3^)	Winter	0.1	0.1	0.00	0.004	0.07	0	0.3	0.3	0.1	0.561	0.561	0
	Summer	0.2	0.2	0.18	0.006	0.08	0.1	0.3	0.2	0.13	0.117	−1.14	0.1
	Rainy	0.2	0.2	0.00	0.005	0.07	0	0.3	0.3	0.1	−0.11	−0.046	0

The descriptive statistics for seasonal variation of ^222^Rn concentration (*C*_*Rn*_) and ^220^Rn concentration (*C*_*Tn*_) in Mansa and Muktsar districts are given in Table 2. It is vivid that the overall mean *C*_*Rn*_ in three seasons was 45 Bq m^−3^ with high ^222^Rn concentration during rainy season (54 Bq m^−3^) and the lowest concentration in summer season (32 Bq m^−3^). The average *C*_*Rn*_ in rainy season is 1.1 fold higher than the winter season. The ratio of rainy to summer ^222^Rn level is 1.7 due to the variation of the soil moisture in different seasons. The soil moisture content is higher in the rainy season as compared to summer or winter season. The rainfall sealing the outer soil surface and elevate the negative pressure field in the room. The negative pressure field generated by the house is responsible for the transportation of soil gas from large distances. The overall mean of *C*_*Tn*_ was found to be 44 Bq m^−3^ across all seasons. The *C*_*Tn*_ was highest during winter season with mean value of 54 Bq m^−3^ and lowest concentration in summers season (34 Bq m^−3^). The seasonal variation of *C*_*Rn*_ is different as compared to *C*_*Tn*_ because of shorter diffusion length and half – life of ^220^Rn. As *C*_*Tn*_ is not affected by ventilation rate^35^, even then *C*_*Tn*_ is higher in the winter season as compared to rainy and summer seasons.

**Figure 7:**
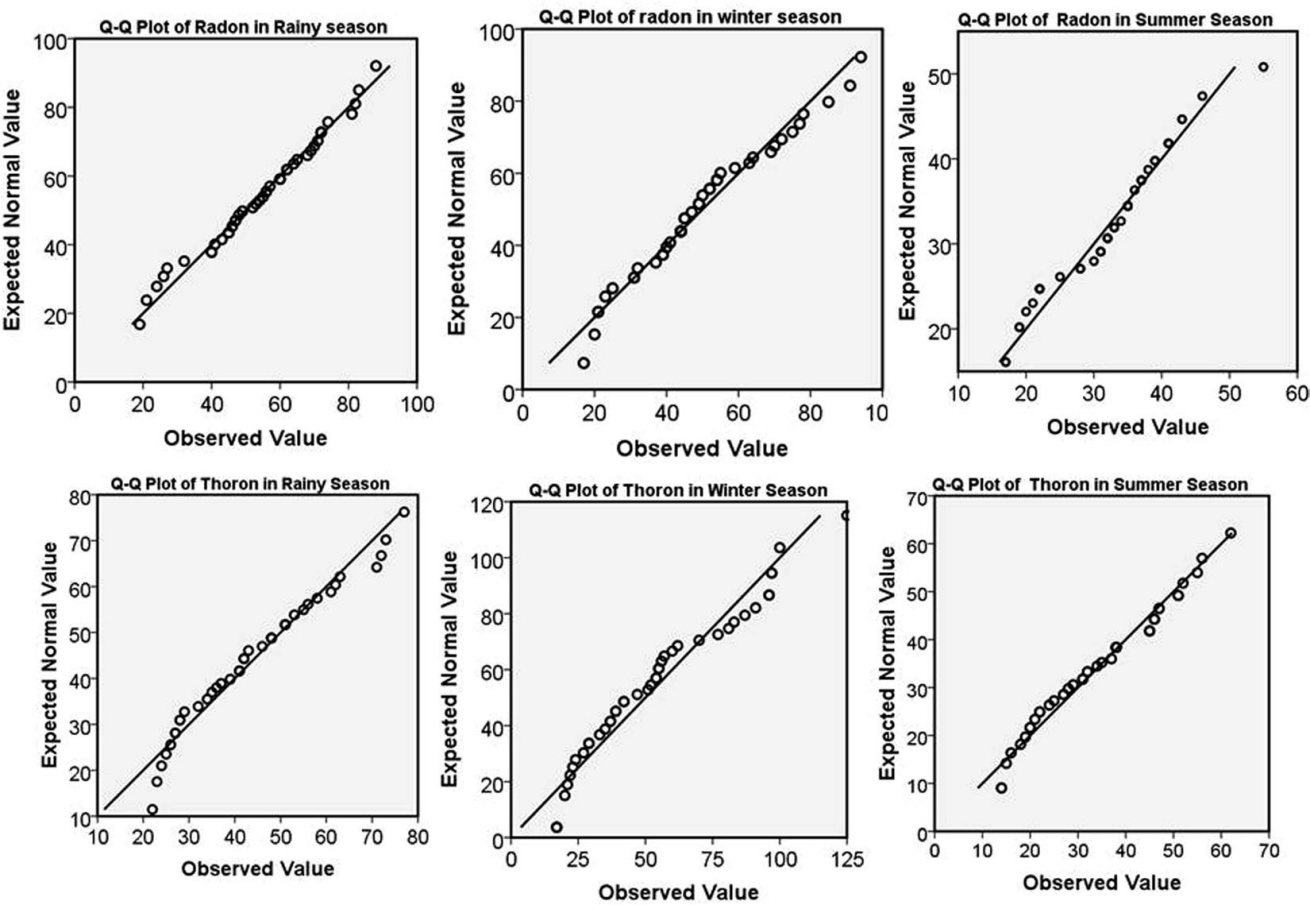
Q-Q Plots for ^222^Rn and ^220^Rn.

The mean and median values of ^222^Rn and ^220^Rn are indicated that the distribution is normal and can be justified by skewness (S_K_), kurtosis values (range between −2 and +2) as given in Table 2. The Q -Q plots (probability plots) have been used to confirm the statistical distribution of data around their mean. Figure 7 demonstrates normal Q-Q plots of *C*_*Rn*_ and *C*_*Tn*_ during rainy, winter and summer seasons. During rainy season, ^222^Rn has normal distribution (S_K_ = −0.21) while ^220^Rn is rightly skewed (S_K_ = 0.49). In winter season, both ^222^Rn (S_K_ = 0.42) and ^220^Rn (S_K_ = 0.61) appears to be rightly skewed, with more variation seen in ^220^Rn distribution. In summer season, ^222^Rn has normal spread with an outlier (S_K_ = −0.01), however, ^220^Rn is rightly skewed (S_K_ = 0.21). The results from these Q-Q plots are corroborating with box-plots. The Q-Q plots of *C*_*Rn*_ and *C*_*Tn*_ lie on a straight diagonal line with minimal deviations indicating normality. Tests of normality (Kolmogorov-Smirnov test) showed that *C*_*Rn*_ and *C*_*Tn*_ in three seasons follow normal distributions and have a further possibility to proceed with parametric statistics.

Figures 5 and 6 revealed the box-plots of *C*_*Rn*_ and *C*_*Tn*_ in three seasons (range of data, R) and it has been found that variation in *C*_*Rn*_ was less as compared to *C*_*Tn*_ as indicated by size of the boxes. ^222^Rn has less spread of data than ^220^Rn, with smallest box being that of summer season (IQR = 10, R = 38) and ^220^Rn has widest distribution during winter season (IQR = 49, R = 108). The only one outlier in the overall data has been found in summer season for ^222^Rn. The median values of *C*_*Rn*_ in three seasons are nearly in the middle of their boxes. For *C*_*Rn*_ the median values are shifted towards 3^rd^ and 1^st^ quartiles in summer and winter seasons, respectively. However, the amount of data on both sides of the boxes is largely unequal for *C*_*Tn*_ than *C*_*Rn*_.

### Seasonal Variation for EEC of ^222^Rn and ^220^Rn progeny

Unlike their parent nuclides, the seasonal demeanor of progenies for ^222^Rn and ^220^Rn has shown distinct consequences in the studied region as given in Table 2. During the rainy season, the average EERC_A+U_ and EETC_A+U_ were 22 Bq m^−3^ and 1.7 Bq m^−3^. In winter and summer seasons, the average EERC_A+U_ and EETC_A+U_ were 24 Bq m^−3^, 1.8 Bq m^−3^ and 15 Bq m^−3^, 1.6 Bq m^−3^ respectively. In winter season the average EERC_A_ and EETC_A_ (Equilibrium equivalent concentration of attached ^220^Rn progeny) in the studied region was 22 Bq m^−3^ and 1.6 Bq m^−3^ respectively. During summer and rainy season, the average EERC_A_ and EETC_A_ (Equilibrium equivalent concentration of attached ^220^Rn progeny) was 15 Bq m^−3^, 1.5 Bq m^−3^ and 20 Bq m^−3^, 1.7 Bq m^−3^ respectively.

**Figure 8:**
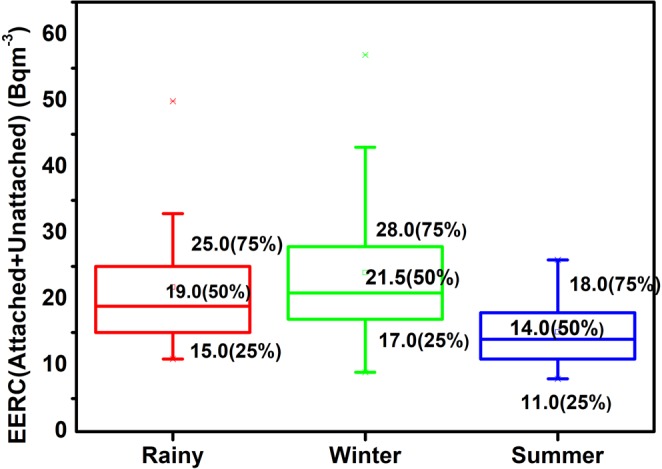
Seasonal Variation of EEC of ^222^Rn.

**Figure 9:**
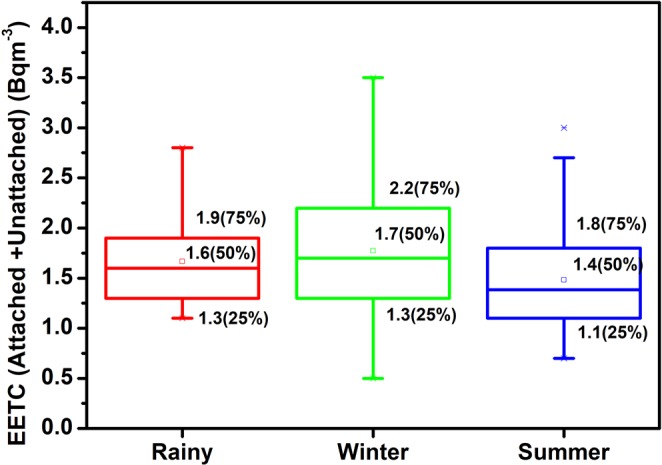
Seasonal Variation of EEC of ^220^Rn.

**Figure 10:**
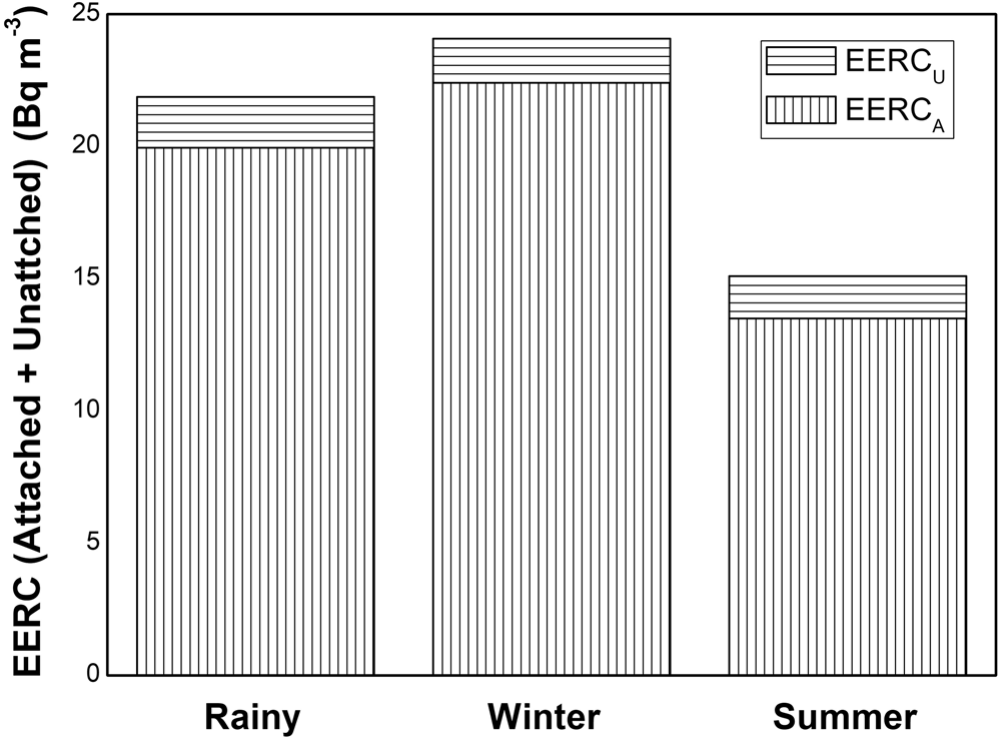
Attached and unattached seasonal variation of EEC of ^222^Rn.

**Figure 11:**
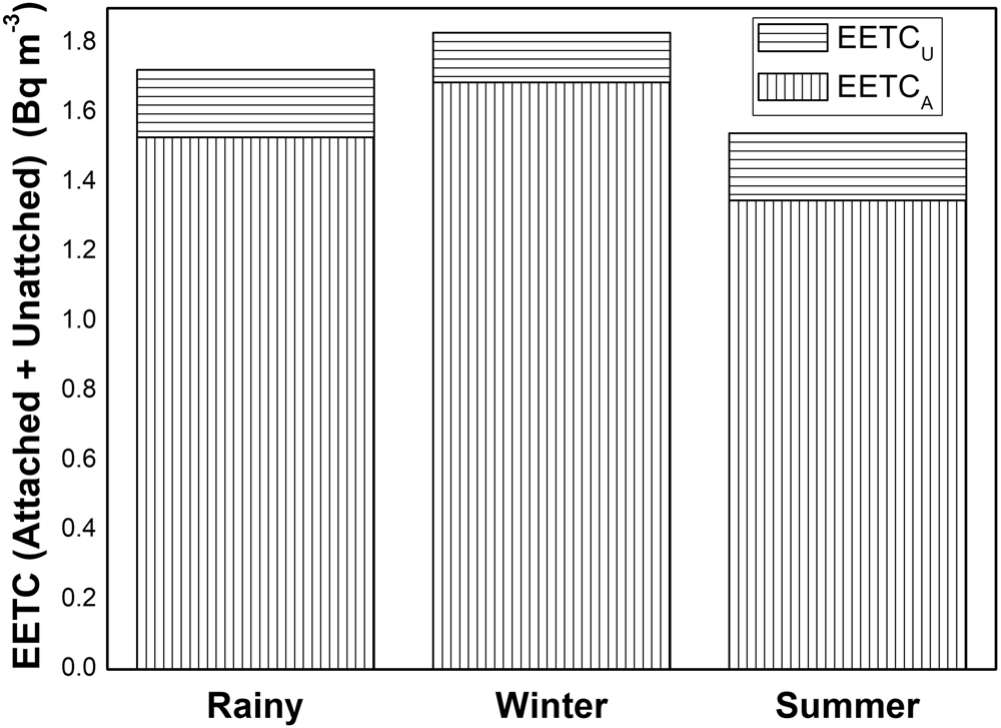
Attached and unattached seasonal variation of EEC of ^220^Rn.

Statistically, the 5% trimmed mean value and the mean value of EERC in rainy, winter and summer season indicated no outliers (<Q1–1.5 * IQR; >Q3 + 1.5 * IQR) in the data. The EETC also has a normal distribution in different seasons as explained by descriptive statistics (S_k_, K and IQR) in Table 2. The box-whisker plots revealed more data spread in EERC as compared to EETC (Figs 8 and 9). In general, 95% attached EEC present in the indoor atmosphere and the seasonal behaviour of total EEC (EERC_A+U_ and EETC_A+U_) relies on attached EEC. The EERC_A_ was greater in winter season as compared to rainy or summer season due to the poor ventilation in the winter season and faster formation process of attached progeny aerosols as shown in Figs 10 and 11. The aerosol concentration increases with the decrease of temperature in the winter season (India)^36^. The higher aerosol concentration in winter season tends to increase the ^222^Rn and ^220^Rn attached progeny concentration in the winter season. However, a seasonal pattern for EETC_A+U_ and EETC_U_ has been not observed in the studied region.

Pearson correlation analysis has been performed to determine the interrelation between the ^222^Rn, ^220^Rn and their attached/unattached EEC in different seasons. A significant and positive correlation has been observed between ^222^Rn, ^220^Rn and different seasons respectively (Table 3). This suggests that ^222^Rn and ^220^Rn separately varied during three seasons with no impact on each other and have an influence on their respective concentrations. ^222^Rn concentrations during the three seasons was significantly associated with attached and unattached EEC of ^222^Rn (r = 0.37–0.72, p < 0.05). However, there was no relationship between ^220^Rn and EEC of ^220^Rn.

**Table 3:** Correlation coefficient of ^222^Rn and ^220^Rn activity concentration among different seasons.

	Rainy-Winter	Rainy-Summer	Winter-Summer
^222^Rn	0.796	0.757	0.644
^220^Rn	0.762	0.814	0.876

### Seasonal variation of Equilibrium factor for ^222^Rn and ^220^Rn

From the last few decades, UNSCEAR specified the value of 0.4 for ^222^Rn and 0.02 for ^220^Rn^37,38^. For an accurate dose assessment, it is necessary to estimate the equilibrium factor of each dwelling due to different ventilation conditions and building material. The equilibrium factor for ^222^Rn (*F*_*Rn*_) and ^220^Rn *(F*_*Tn*_) has been calculated season- wise. The F_*Rn*_ during rainy, winter and summer seasons was 0.42 ± 0.15, 0.51 ± 0.14 and 0.49 ± 0.15 respectively. It has been observed that F_Rn_ was lower in the rainy season due to higher C_Rn_ and lower EERC_A+U_ in the same season. The overall arithmetic means of F_Rn_ and F_Tn_was 0.47 ± 0.04 and 0.05 ± 0.01, respectively. The F_Tn_during rainy, winter and summer seasons was 0.04 ± 0.03, 0.05 ± 0.03 and 0.05 ± 0.04, respectively. Due to the much smaller value of F_Tn_, a proper relation has not been observed seasonally. However, F_Rn_ varies in different indoor environments due to variation in environmental parameters (ventilation rate, temperature etc). Further, it is worth highlighting that F_Tn_ varies in the same indoor environment due to the fact of ^220^Rn gas is not uniformly distributed in a room but ^220^Rn progeny having relatively high half-life is uniformly distributed^39^. Hence, it is problematic to define season wise F_Tn_ due to the difference in physical properties of ^220^Rn and its progeny.

### Seasonal variation of Unattached Fraction for ^222^Rn (${{\boldsymbol{f}}}_{{\boldsymbol{p}}}^{{\boldsymbol{R}}{\boldsymbol{n}}}$), ^220^Rn (${{\boldsymbol{f}}}_{{\boldsymbol{p}}}^{{\boldsymbol{T}}{\boldsymbol{n}}}$)

Most of the unattached ^222^Rn and ^220^Rn progeny is deposited in the respiratory tract during breathing whereas 80% of the attached progenies get exhaled without deposition^40^. So, the estimation of unattached fraction is necessary for accurate dose assessment. The unattached fractions for ^222^Rn (${f}_{p}^{Rn}$) and ^220^Rn (${f}_{P}^{Th}$) have been calculated (using Eq. 12, 12a) season wise.

12 $${f}_{p}^{Rn}=\frac{EER{C}_{U}}{EER{C}_{A+U}}$$

12a $${f}_{P}^{Tn}=\frac{EET{C}_{U}}{EET{C}_{A+U}}$$

It has been found that unattached fraction is slightly higher in summer season as compared to winter and rainy seasons. However total EERC_A+U_ and EETC_A+U_ were higher in the winter season, but ${f}_{p}^{Rn}$ and ${f}_{p}^{Tn}$ are lower in the winter season. In the winter season, the aerosol concentration is higher which is attributed to high values of attached progeny in the dwellings. The aerosol concentration decreases during rainfall in India^36^. The EECs for ^222^Rn and ^220^Rn are lower in summer season due to good ventilation and higher exchange rate between indoor and outdoor environments. The overall arithmetic means (rainy, winter and summer seasons) of ${f}_{p}^{Rn}$ and ${f}_{p}^{Tn}$ were 0.09 ± 0.02 and 0.10 ± 0.03, respectively. A relation ${f}_{p}^{Rn}=400/Z$ has been used to calculate the aerosol concentration (Z) from the unattached fraction of ^222^Rn for the estimation of attachment coefficient^21,41^. In the past studies, the aerosol concentration for a residential environment was considered to be 10,000 *cm*^−3^ with ${f}_{p}^{Rn}=0.07$ for an indoor environment^42^. UNSCEAR suggested a central value of 0.05 (in homes) for *f*_*p*_ and it can vary by a factor of 2 depending on air filtration and local source^15^. The observation for ${f}_{p}^{Rn}$ in the present manuscript is in agreement with literature (Table 4).

**Table 4:** Comparison of *F*_*Rn*_, ${f}_{p}^{Rn}$ and *β* with previously published values.

Publication	Environment Status	*β*(*cm*^3^*h*^−1^)	${{\boldsymbol{f}}}_{{\boldsymbol{p}}}^{{\boldsymbol{R}}{\boldsymbol{n}}}$	F_Rn_
ICRP^21^	Indoor	………..	0.08	0.4
Jilek *et al*.^51^	Indoor air	5 × 10^−3^	0.08	0.4–0.5
El- Hussein *et al*.^52^	Candlelight	0.46 × 10^−3^	0.019	0.34
El- Hussein *et al*.^52^	Cigarette	11.4 × 10^−3^	0.01	0.32
Porstendoefer^53^	Indoor air	5 × 10^−3^	0.05–0.1	0.25–0.4
Porstendorfer and Mercer^54^	Room Aerosol	4.3 × 10^−3^	………..	……..
Present work	Indoor air	9.6 × 10^−3^	0.09 ± 0.02	0.44 ± 0.09

### Variation of attachment rate (*X*_*Rn*_) and attachment coefficient (β) of ^222^Rn

**Figure 12:**
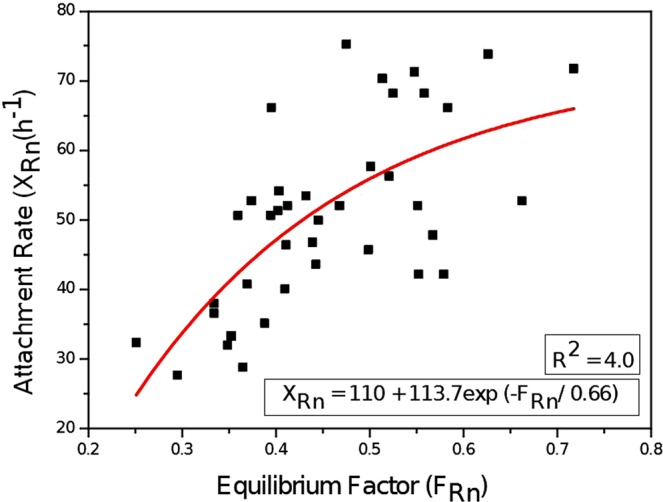
Relation between X_Rn_ (h^−1^) and F_Rn_.

While studying the seasonal behaviour of attached and unattached progeny, it is necessary to discuss the attachment rate and attachment coefficient. The *X*_*Rn*_ and *β* have been calculated using Eqs (5–10). The *X*_*Rn*_ in the studied area is 64 *h*^−1^ in winter, 38 *h*^−1^ in summer and 47 *h*^−1^ in rainy season. Attachment coefficient is defined as the attachment rate per unit aerosol concentration. In past studies, the ratio ${X}_{Rn}/Z$ was denoted as the average attachment coefficient (β)^34^. The attachment rate coefficient increases with AMAD (Activity median aerodynamic diameter) and larger AMAD describes aerosols with a larger diameter which induce increasing attachment rate^42^. The value of *β* is slightly greater in winter (0.010 *cm*^3^*h*^−1^) than summer (0.009 *cm*^3^*h*^−1^). The average calculated attachment rate coefficient is greater than 0.005 *cm*^3^*h*^−1^ as reported by Porstendorfer^43^ and almost equal to 1.45 × 10^−9^ *m*^3^*h*^−1^ given by Stevanovic *et al*.^44^. The average estimated *F*_*Rn*_, *X*_*Rn*_ and *β* are concerned as per the values given by other investigators in Table 4. A positive correlation (R^2^ = 0.4) has been observed between *F*_*Rn*_ and *X*_*Rn*_ (*h*^−1^) and the best fit is shown in Fig. 12. A correlation between ${f}_{p}^{Rn}$ and *F*_*Rn*_ has been already studied in the past studies^45,46^.

### Estimation of absorbed dose rate {*D*_*ab*_(*nGyh*^−1^)} and Effective dose

As ICRP adopted DCF’s of 5 mSv WLM^−1^ and 4 mSv WLM^−1^ for homes and mines and later recommended 3.8 mSv WLM^−1^ ^16^. The variation of DCF’s for ^222^Rn, estimated from epidemiological studies and dosimetric approach varied from 3.8–9.5 mSv WLM^−1^ ^16^. The dosimetric evaluation of the absorbed dose to the bronchial epithelium per unit exposure varied from 5–25 nGy (Bq h m^−3^)^−1^ with an average central value of 9 nGy (Bq h m^−3^)^−1^ reported by UNCEAR^15^. In the present manuscript, direct energy deposition rate (alpha fluence), D_ab_ (*nGyh*^−1^) and effective dose have been estimated from total EEC. The energy deposition rate in bronchial epithelium has been calculated using Eq. (13).

13 $${D}_{ab}(nGy{h}^{-1})=\frac{{\rm{\Phi }}({\rm{\alpha }}c{m}^{-2}{s}^{-1})\times {\rm{Energy}}({\rm{Mev}}\,{{\rm{\alpha }}}^{-1})\times 1.6\times {10}^{-6}({\rm{erg}}{({\rm{Mev}})}^{-1}}{{R}_{t}(cm)\times {\rho }_{t}(gram{(cm)}^{-3})\times 100(erg{(gram)}^{-1})/rad}$$

Where *Φ* (α*cm*^−2^*s*^−1^) is an alpha flux, *R*_*t*_ = Range of α- Particle in tissue, ρ_t_ = Density of tissue. A 50% of this flux has been taken due geometry consideration of basal and secretory cells. For the indoor environment, an occupancy factor of 0.8 has been used to calculate the total dose^16^.

The absorbed dose rate for lungs (*D*_*lung*_(*nGy h*^−1^)), for trachea- bronchial region (*D*_*TB*_(*nGy h*^−1^)) and for the pulmonary region (*D*_*P*_(*nGy h*^−1^)) have been estimated for comparison using UNSCEAR report using Eqs (14–16) ^47^.

14 $${D}_{lung}(nGy\,{h}^{-1})=0.04\times {222}_{Rn}(Bq{m}^{-3})$$

15 $${D}_{TB}(nGy\,{h}^{-1})=7\times EERC(Bq{m}^{-3})$$

16 $${D}_{P}(nGy\,{h}^{-1})=0.9\times EERC(Bq{m}^{-3})$$

17 $$F={C}_{Rn}\times \frac{{F}_{Rn}}{3700}\times \frac{time}{170}\times DCF$$

The *D*_*ab*_(*nGyh*^−1^) in the studied area varied from 32 *nGyh*^−1^ to 121 *nGyh*^−1^ with an average value of 60 *nGyh*^−1^. In UNSCEAR 1988 report^47^, the *D*_*lung*_(*nGyh*^−1^) was estimated from ^222^Rn, *D*_*TB*_(*nGyh*^−1^) and *D*_*P*_(*nGyh*^−1^) was estimated from EEC as given in Eqs (14–16) respectively. These results showed a huge gap between these two approaches and in the same report, it was further assumed that bronchial dose is better related to ^222^Rn gas concentration directly instead of from total EEC. However, in the later reports of UNSCEAR, a previous DCF of 5.7 mSv WLM^−1^ (based on EEC) has been considered for the estimation of radiological dose. In the present manuscript, *D*_*ab*_(*nGyh*^−1^) is floating in between both approaches. A comparison between these doses has beengiven in Table 5. The calculated *D*_*ab*_(*nGyh*^−1^) shows similar variance as reported by UNSCEAR (2000) report for indoor gamma exposure rate^6^. The effective dose in the studied area varied from 1.9 mSv a^−1^ to 7 mSv a^−1^ with an average of 3.4 mSv a^−1^ respectively. The calculated effective dose is in consent with past studies and these different doses have been calculated using Eq. (17) from DCFs discussed in different model asreported in Table 6. The calculated dose is greater than epidemiological approach dose and almost similar to the dosimetric dose model (DCF of 15 mSv a^−1^)^48^.

**Table 5:** Absorbed dose rates (D_ab_(*nGyh*^−1^)) in Mansa and Muktsar districts.

	Present Investigation *D*_*ab*_(*nGyh*^−1^)	UNSCEAR 1988 (Based on EEC)^47^			UNSCEAR 1988 (Based on ^222^Rn)^47^ *D*_*lung*_(*nGyh*^−1^)	UNSCEAR 2000 (from Gamma)^6^ *D*(*nGyh*^−1^)
		*D*_*TB*_(*nGyh*^−1^)	*D*_*P*_(*nGyh*^−1^)	Total (*nGyh*^−1^)		
Min	32	77	10	87	0.4	20
Max	121	289	37	327	2.9	200
Avg	60	142	18	161	1.8	84

**Table 6:** Estimation of effective dose in Mansa and Muktsar districts.

	Absorbed Dose (Gray) × 10^−5^	Effective Dose mSv	ICRP 39^55^ mSv	ICRP 65, 103^16,18^ mSv	Marsh *et al*.^56^ mSv	Nikezic *et al*.^48^ mSv	UNSCEAR^37^ mSv	Bangotra *et al*.^46^ mSv
Min	11.6	1.9	1.2	0.5	1.6	1.8	0.7	1.3
Max	43.6	7.0	4.6	1.8	5.9	6.9	2.6	4.8
Avg	21.5	3.4	2.3	0.9	2.9	3.4	1.3	2.4

## Conclusion

Different statistical tools used in the present study reveal the normal distribution of ^222^Rn and ^220^Rn in three seasons of a year. The indoor ^222^Rn concentration is the highest in rainy and winter seasons and the lowest in summer season. However, ^220^Rn concentration is the highest in winter season and demonstrated the distinct seasonal behavior a part from that of ^222^Rn. A large spatial variation in the activity concentrations of indoor ^222^Rn was found in Sardoolgarh and Behniwala villages. The activity concentration of indoor ^220^Rn was found relatively high in Kulrian and Ahlupur villages of Mansa district. The activity concentrations of natural radionuclides (^226^Ra and ^232^Th) in soil of these villages are reported to be higher than corresponding world average values of ^226^Ra (35 Bq kg^−1^) and ^232^Th (45 Bq kg^−1^)^2^. The high values of ^226^Ra in soil (or high indoor ^222^Rn concentration) may be attributed to presence of uranium bearing rocks, such as granitic rocks, carbonate sediments and calcareous shales in vicinity of Tosham ring (south of Mansa district)^22–25^. The observed pattern of seasonal variation in different parameters is not in agreement with the past studies (due to imperfect selection of seasons and directly used bare mode LR-115 as detector)^49^. In general, indoor ^222^Rn and ^220^Rn concentration in the studied area are lower than the recommended action level of 200–300 Bq m^−3^ ^7^. The attached EEC of ^222^Rn (EERC_A_) is high in winter season as compared to other seasons due to faster formation process of attached progeny aerosols and poor ventilation in winter.

The calculated values of F_Rn_ (0.47 ± 0.04) and F_Tn_ (0.05 ± 0.01) in the studied area are slightly greater than those given by UNSCEAR and ICRP^21,37,38^. The calculated value of F_Rn_ is slightly higher in summer season as compared to rainy and winter seasons. Due to the very smaller value of F_Tn_, a proper seasonal relation has not been observed for F_Tn_. The overall arithmetic means (rainy, winter and summer seasons) of unattached fraction for ^222^Rn and ^220^Rn (${f}_{p}^{Rn}\,$and $\,{f}_{p}^{Th}$) were 0.09 ± 0.02 and 0.10 ± 0.02, respectively. The ${f}_{p}^{Rn}$ and $\,{f}_{p}^{Th}$ are lower in winter season as compared to rainy and summer seasons due to the higher value of attached EEC present in the winter season. Positive and significant correlations have been observed among equilibrium factor of ^222^Rn (F_Rn_), ${f}_{p}^{Rn}\,$and *X*_*Rn*_ (*h*^−1^). Both ${f}_{p}^{Rn}$ and *X*_*Rn*_ (*h*^−1^) having a converse behaviour with F_Rn_. Equilibrium factor of ^222^Rn (F_Rn_) is an important parameter that have correlation with an unattached fraction (${f}_{p}^{Rn}$), attachment rate (*X*_*Rn*_) and aerosol concentration (Z).

**Figure 13:**
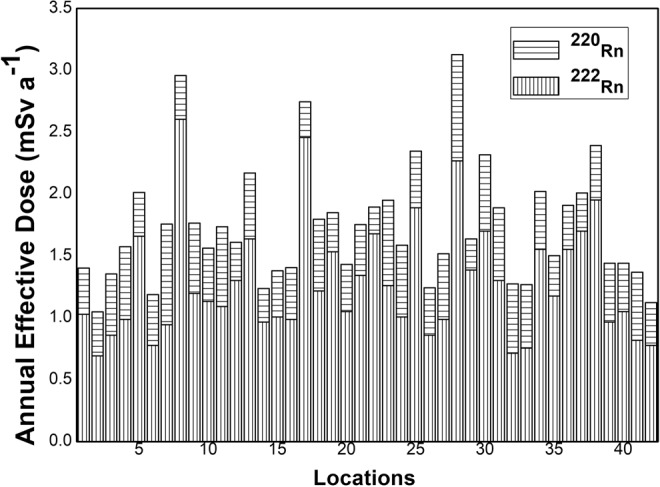
Annual effective doses due to ^222^Rn and ^220^Rn.

The average *D*_*ab*_ (*nGyh*^−1^) (absorbed dose) in the studied area is 60 *nGyh*^−1^ that minimizes the gap to estimate the absorbed dose rate from ^222^Rn and EEC approach, as given in UNSCEAR^15^. The annual effective dose rate in the studied region is 3.4 mSv a^−1^. Figure 13 shows that there is significant contribution of ^220^Rn to total inhalation dose which suggests that simultaneous measurements of ^222^Rn and ^220^Rn are important for an accurate dose assessment. The calculated value of effective dose in the present investigation is greater than that reported by UNSCEAR^37^ and Marsh *et al*.^45^ approach. However, it is almost equal to dose given by Nikezic *et al*.^48^. The estimated dose is greater than average worldwide dose of 1.26 mSv a^−1^ for ^222^Rn^46,50^. The present approach followed dosimetric approach rather than epidemiological approach. The dose estimated in the present study is 3.8 fold higher than epidemiological approach. This discrepancy may be due to the use of revised alpha radiation weighting factor in the previous report of ICRP and mostly dosimetric models except ICRP 24 were developed after 1980^13,14^. The other aspect of this discrepancy may be unregistered cancer cases under epidemiological approach. This discrepancy can be solved to deploy long-term measurements of both approaches in active mines rather to assumed previous epidemiological based unregistered data in official reports.
